# Editorial: From depth (needle) to surface: electromyography as a diagnostic tool in identifying neuromuscular changes associated with neurological disorders

**DOI:** 10.3389/fnhum.2023.1214106

**Published:** 2023-06-07

**Authors:** Rajat Emanuel Singh, Kamran Iqbal, Jongsang Son

**Affiliations:** ^1^Department of Kinesiology, Northwestern College, Orange City, IA, United States; ^2^School of Engineering and Engineering Technology, University of Arkansas at Little Rock, Little Rock, AR, United States; ^3^Department of Biomedical Engineering, New Jersey Institute of Technology, Newark, NJ, United States

**Keywords:** stroke, electromyography, motor module, MScanFit, upper limb, mirror movement

Neurological disorders are the major cause of disability-adjusted life years (DALYs) and the second leading cause of death worldwide (GBD, 2019). These disorders are generally associated with anatomical and physiological abnormalities in the peripheral nervous system (PNS) and/or central nervous system (CNS). Previous studies focused on scoring systems and experimental designs to measure the improvements in sensorimotor functions associated with changes in neural pathways during rehabilitation therapy. However, it is very challenging to measure and account for neurophysiological changes associated with rehabilitation therapies using a scoring system only as the circuits constituting CNS and PNS are bioelectrical in nature. In the past few decades, electromyography (EMG) has gained strong attention in rehabilitation sciences among physical therapists, occupational therapists, neuroscientists, biomedical engineers, and bio-mechanists. The EMG device assists in the acquisition of the electrical activity of the muscles invasively through needle EMG and non-invasively through surface EMG (sEMG). Considered to be the superposition of motor unit action potential, EMG signals display motor output which correlates well with force production, the mechanics of limbs, and the synergistic activation of muscles. Thus, EMG signals may better describe the neuromuscular and/or neuromechanical mechanisms associated with neurological disorders.

The objective of this Research Topic is to compile a collection of scholarly articles that offers insight into the spinal and supraspinal circuits of patients with neuromuscular disorders. These articles should present various computational, theoretical, and experimental models to aid in the effective management of neuromuscular disorders, such as strokes, using EMG-based rehabilitative devices. Therefore, we invited researchers from the field of biomedical engineering, biomechanics, neuroscience, physical therapy, etc. to contribute to this issue. In response to the call for contributions, three articles and a brief research report were submitted and accepted. We have outlined a summary of each article published in this Research Topic below.

Seo et al. decomposed multichannel EMG signals from the upper extremity to examine motor modules' contribution to exploratory isometric forces among stroke patients during a three-dimensional target force task. The motor modules also known as muscle synergies are the building blocks of movement, and the CNS selects a small dimension of motor modules from a high-dimensional space to form a movement (Singh et al., 2018). In this study, they found that while the motor modules were similar across stroke and control participants, the composition of muscles within the shoulder adductor/flexor motor modules displayed abnormal activation of the pectoralis, anterior, and medial deltoid muscles in stroke patients. Additionally, each motor module's activation profiles were modulated in both groups to meet the task demands, with stroke patients displaying more peculiar characteristics. The modulation in activation profiles of stroke patients was attributed to a narrow and skewed force space. This has been validated with the implementation of principal component analysis and multivariate multiple linear regression analysis on concatenated motor modules' activation profiles and exploratory force data, which revealed that the abnormality in shoulder muscle activation of stroke patients results in constrained and feasible endpoint forces. The control group did not exhibit this motor behavior. Therefore, the decomposition of multivariate EMG signals revealed that altered motor modules play a role in the limited and feasible range of endpoint forces.

Furthermore, abnormality in the activation of shoulder muscles can lead to functional impairments in the hand muscles. Phan et al. conducted a pilot study, which demonstrated these results. During a seated task that involved transporting a cylinder at varying kinematic positions, stroke patients exhibited abnormal kinematics and muscular coordination of shoulder (proximal) and hand (distal) muscles compared to the control group. In particular, the stroke group displayed a significantly smaller hand aperture compared to the control group during shoulder abduction or elbow extension movement. This constrained functional mechanical behavior observed in stroke patients was due to the co-contraction of hand muscles, which is caused by increased neural coupling between the shoulder and hand muscles. This phenomenon is revealed as high inter-muscular coherence in hand muscles, as computed from the sEMG. Hence, abnormal activation of proximal muscles during seated reaching affects the muscle coordination and kinematics of the distal region.

The research article by Dai et al. explores the relationship between the emergence of mirror movements and the quality of motor function in stroke patients. Mirror movements are involuntary movements occurring on one side of homologous muscles when unilateral voluntary movements are performed. sEMG techniques revealed mirror activities in both affected and unaffected muscles during maximal contractions. The authors used standardized net excitation (SNE) and overflow percentage (OF) to measure the strength of mirror activities. The authors found that OF in the case of unilateral contraction tasks on the affected side was significantly higher than unilateral contraction tasks on the unaffected side. The SNE appeared unrelated to the motor function of the patients but had a positive relationship with the improvement of motor function, indicating that the activation of the contralateral cortex may be involved in the potential recovery of motor function of the affected side. Hence, the high OF on the contralateral side due to the stroke resulted in highly asymmetric mirror movement.

The brief research report by Song et al. explores the efficacy of the MScanFit program to estimate the number of motor units based on compound muscle action potential (CMAP) from the recordings of five hand muscles, i.e., first dorsal interosseous, abductor pollicis brevis, abductor digiti minimi, second lumbrical, and abductor hallucis muscles. The authors found that their motor unit number estimates were mostly consistent with previous studies. These findings suggest that the technique may be a promising tool for early diagnosis and monitoring of neuromuscular disorders.

In summary, our Research Topic has successfully provided valuable information on the characteristics of neuromuscular disorders, particularly strokes, at the CNS level. Furthermore, it has provided effective monitoring of neuromuscular disorders. For example, MScanFit is beneficial for tracking the loss and reinnervation of motor units at different stages of stroke rehabilitation. Additionally, our topic offers therapeutic solutions for practitioners. For instance, therapy-based training involving seated reaching tasks improves motor function. Therefore, the markers identified in these studies are necessary for rehab practices. Each of these articles provides experimental, theoretical, and computational solutions for the rehabilitation of patients afflicted with neuromuscular disorders, as shown in Table 1.

**Table 1:** Summary of Research Topic experimental, theoretical, and computational approaches.

**Author**	**Experimental**	**Theoretical**	**Computational**
Seo et al.	Seated reaching task with multiple degrees of freedom	Muscle synergy theory: Modulation in activation profiles causes endpoint force abnormalities	Time series decomposition using factorization algorithms, such as NNMF and PCA
Phan et al.	Seated reaching task with multiple degrees of freedom	Reduction in degrees of freedom is due to increased intermuscular coherence	Time and Frequency domain analysis of sEMG signal features
Dai et al.	Comparative analysis of contralateral and ipsilateral side during unilateral isometric contraction	The existing mirror movements on both sides are highly asymmetric due to the ipsilateral side being afflicted with a stroke	Statistical analysis of sEMG using SNE and OF
Song et al.	NA	NA	Computational comparison of experimental CMAP vs. MScanFit program device

## Author contributions

RES, KI, and JS: writing, drafting, and editing. All authors contributed to the article and approved the submitted version.
